# Echtyma contagiosum in a goat farmer^☆^

**DOI:** 10.1016/j.abd.2026.501376

**Published:** 2026-06-18

**Authors:** Régis Lambrecht Andrade, Florisberto Lambrecht, Aline Paganelli, Hiram Larangeira de Almeida

**Affiliations:** a Medicine Graduation, Universidade Católica de Pelotas, Pelotas, RS, Brazil; b Hospital de Caridade de Canguçu, Canguçu, RS, Brazil; c Pathology Laboratory, Centro de Anatomia Patológica, Pelotas, RS, Brazil; d Post-Graduation Program in Health, Universidade Católica de Pelotas, Pelotas, RS, Brazil

*Dear Editor,*

*Echtyma Contagiosum* (EC), or Orf, is a zoonosis with worldwide distribution caused by the Orf Virus (OrfV), of the genus *Parapoxvirus*.^1^, ^2^, ^3^ The virus is transmissible to humans from infected small ruminants, predominantly sheep and goats. In infected animals, the virus manifests as proliferative lesions frequently appearing on the lips, muzzle, ears, eyelids, and nostrils, and less commonly on the udder, genitalia, and feet.^1^

Due to its self-limited nature, treatment is not necessary in immunocompetent patients with a solitary lesion; however, immunocompromised individuals have an increased risk of developing large and persistent lesions. Immunity against the virus is short-lived, and reinfection may occur.^3^

A 46-year-old female rural worker sought medical care due to a painful ulceration on the right thumb, measuring 1.6 × 1.0 cm, as well as a small erythematous-edematous lesion with a central crust at the base of the right index finger (Fig. 1A). She reported that both lesions had appeared simultaneously seven days earlier and suspected a spider bite. When questioned about potential contact with animals, the patient stated that she had cared for goats with crusted lesions on their lips (Fig. 2).

**Fig. 1:**
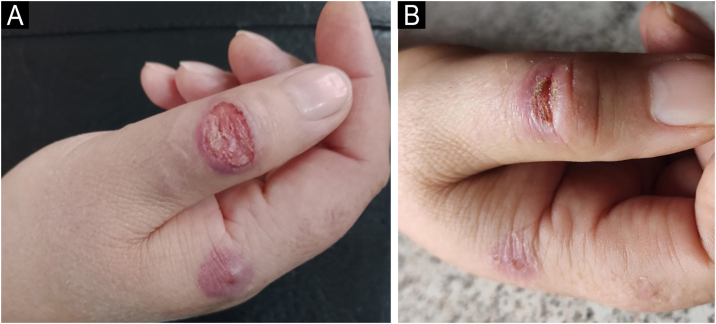
Clinical aspect. (A) Ulceration on the right thumb and a small erythematous-edematous lesion with a central crust at the base of the right index finger. (B) Partial involution after 15-days.

**Fig. 2:**
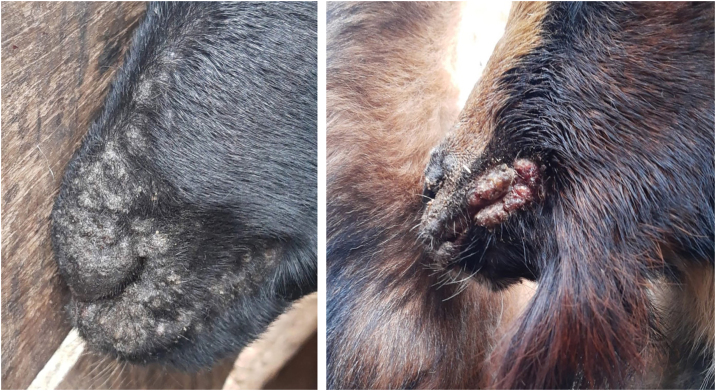
Peri-oral lesions on two goats.

Laboratory tests were performed, including complete blood count, ESR, C-reactive protein, VDRL, urea, and hand radiograph ‒ all of which were normal.

Histopathology of the affected skin revealed a central area of ulceration covered by a scale-crust with compact hyperkeratotic epidermis containing eosinophilic inclusion bodies, ballooning degeneration, and neutrophil collection. The dermis exhibited moderate edema and a dense infiltrate composed of lymphocytes, plasma cells, and occasional neutrophils (Fig. 3). Immunohistochemistry (Fig. 4) was positive for CD2 (T-cell and NK-cell marker), CD4 (T-helper marker), and CD5 (T-cell marker), showed weaker staining for CD8 (regulatory T-cell marker), and was strongly positive for CD68 (monocyte and macrophage marker). It was negative for CD20 (B-cell marker).

**Fig. 3:**
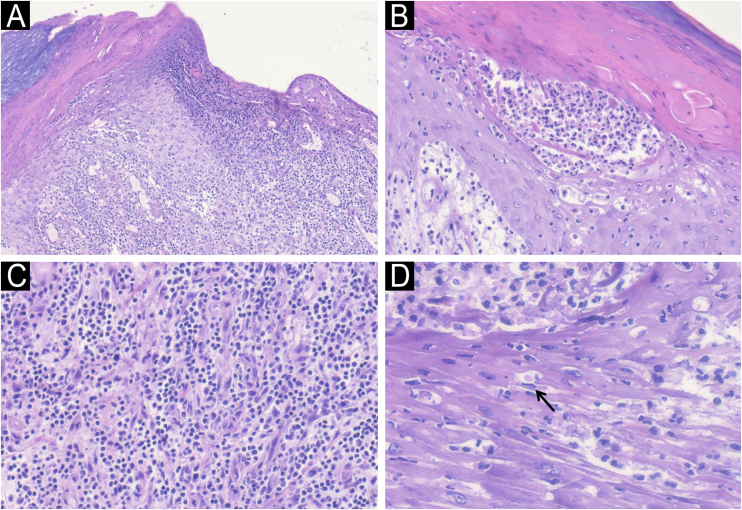
Light microscopy. (A) Central area of ulceration covered by a scale-crust. (B) Neutrophils microabcess. (C) Dense dermal infiltrate (D) Eosinophilic inclusion body (arrow). (Hematoxylin & eosin, ×100, ×400, ×400, ×500).

**Fig. 4:**
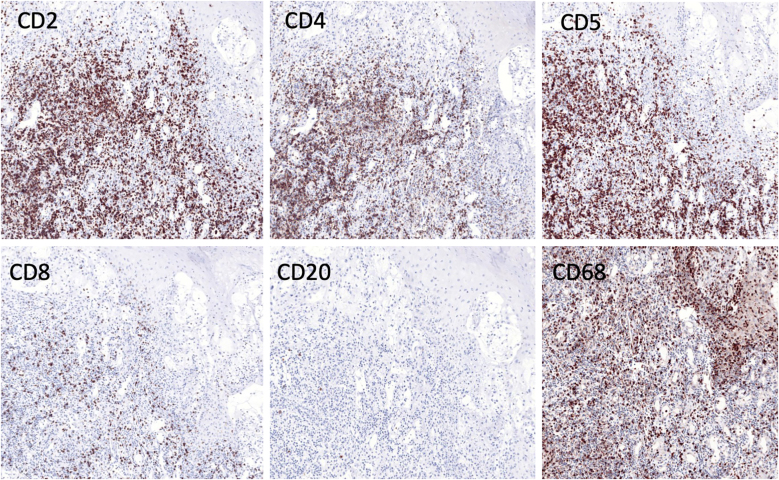
Immunohistochemistry strongly positive for CD2, CD4, CD5 and CD68, weaker staining for CD8 and negative for CD20 (×200).

Since there was no contact with cattle, the milker’s nodule was excluded, and a diagnosis of echtyma contagiosum was established. The lesions regressed spontaneously in three weeks (Fig. 1B), leaving mild residual erythema.

EC is an occupational zoonosis with worldwide distribution.^4^ The OrfV has affinity for domestic ruminants, such as sheep and goats; however, cases have also been reported in wild species.^4^ In animals, lesions are crusted and proliferative, affecting the skin and mucocutaneous junctions. The virus is highly contagious among animals. It is known to tolerate inactivation in dry environments and has been recovered from crusts after several months.^1^ It survives for almost one month in wool and skin after clinical improvement.^1^

Transmission of OrfV to humans may occur through direct contact with an infected animal or via fomites. Most cases are observed in individuals with occupational exposure to animals, such as livestock workers. However, cases have been reported among shepherds, butchers, slaughterhouse workers, handlers of animal hides and wool, veterinarians, zoo visitors, hunters, and participants in religious ceremonies involving animal sacrifice.^5^ The incubation period is 3–7 days,^1^, ^2^ and may reach up to six weeks depending on the severity and immune status of the patient.^4^

The clinical diagnosis of EC depends on the characteristic appearance of the lesions, histopathological features, exposure to animals, and lesion evolution; all of which were typical in this case. Confirmation may be obtained via electron microscopy, PCR, or detection of specific antibodies, which were not available in our setting. There are a few reports of human cases in Brazil,^6^ although many cases in animals have been published.^7^, ^8^

Histopathological findings may vary depending on the stage of the lesion. Early stages are characterized by spongiform degeneration of the epidermis, with variable vesiculation of the superficial epidermis and eosinophilic cytoplasmic inclusion bodies in keratinocytes. Our immunohistochemical findings demonstrated the involvement of T-lymphocytes and histiocytes in the inflammatory response.

In the differential diagnosis, the main entity to be considered is milker’s nodule, also caused by a *Parapoxvirus*, with similar clinical and histopathological features.^9^, ^10^ However, the host animal in that condition is cattle. Neutrophilic dermatoses can also resemble Orf nodules, especially pyoderma gangrenosum and neutrophilic dermatosis of the dorsal hands.

Infection with the OrfV does not result in long-lasting immunity, and individuals may be infected multiple times throughout life; however, subsequent infections may be less pronounced and heal more rapidly or remain unnoticed.^1^

Because animals are the primary reservoirs and sources of infection in humans, preventive measures should focus on vaccination of animals (there is no vaccine for humans) and hand protection during animal handling.^1^

## CRediT authorship contribution statement

Régis Lambrecht Andrade: Study concept and design; data collection, analysis and interpretation of data; writing of the manuscript or critical review of important intellectual content; critical review of the literature, final approval of the final version of the manuscript.

Florisberto Lambrecht: Study concept and design; data collection, or analysis and interpretation of data; writing of the manuscript or critical review of important intellectual content; critical review of the literature, final approval of the final version of the manuscript.

Aline Paganelli: Data collection, or analysis and interpretation of data; writing of the manuscript or critical review of important intellectual content; critical review of the literature; final approval of the final version of the manuscript.

Hiram Larangeira de Almeida Jr: Study concept and design; data collection, or analysis and interpretation of data; writing of the manuscript or critical review of important intellectual content; critical review of the literature, final approval of the final version of the manuscript.

## Financial support

None declared.

## Research data availability

Does not apply.

## Conflicts of interest

None declared.
